# Supplementary material for: Valorization of Different Fractions from Butiá Pomace by Pyrolysis: H2 Generation and Use of the Biochars for CO2 Capture
Source: Molecules. 2022 Nov 3;27(21):7515. doi: 10.3390/molecules27217515 (PMC9658530; doi:10.3390/molecules27217515)
Supplement: Supplementary file 1 [file molecules-27-07515-s001.zip › molecules-1967037-supplementary.pdf]

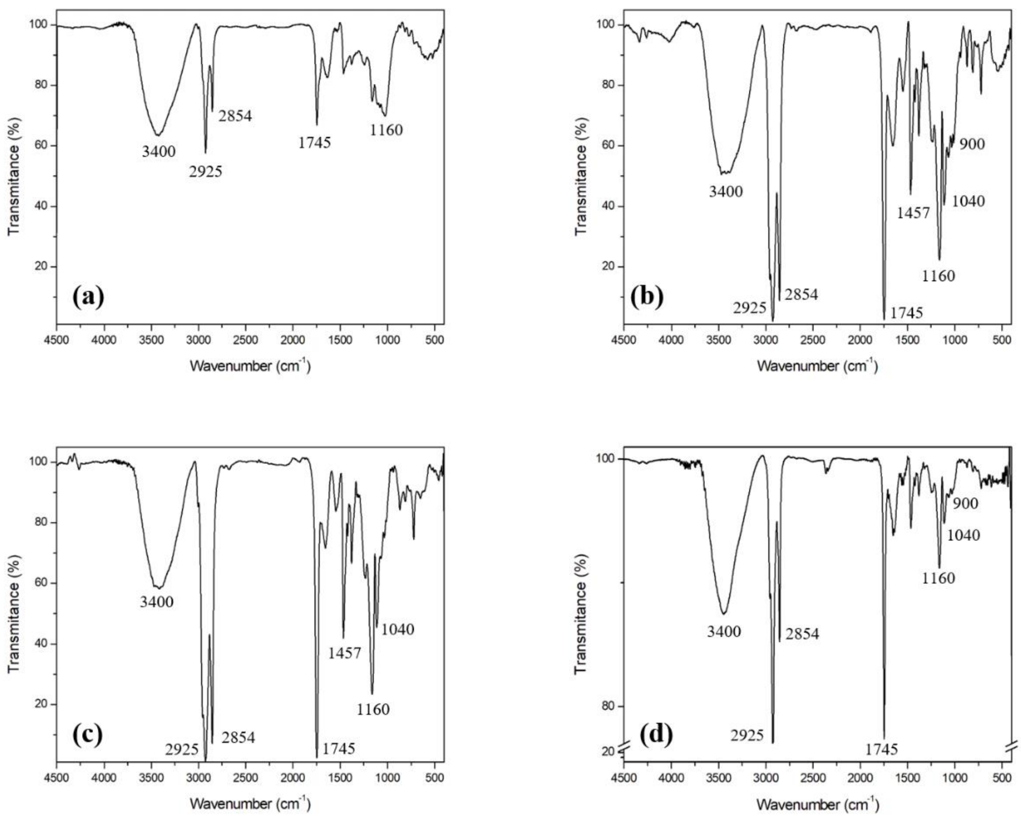

Figure S1. FTIR vibration spectra of butiá precursors (a) FIB, (b) ALM, (c) END, and (d) DOA.

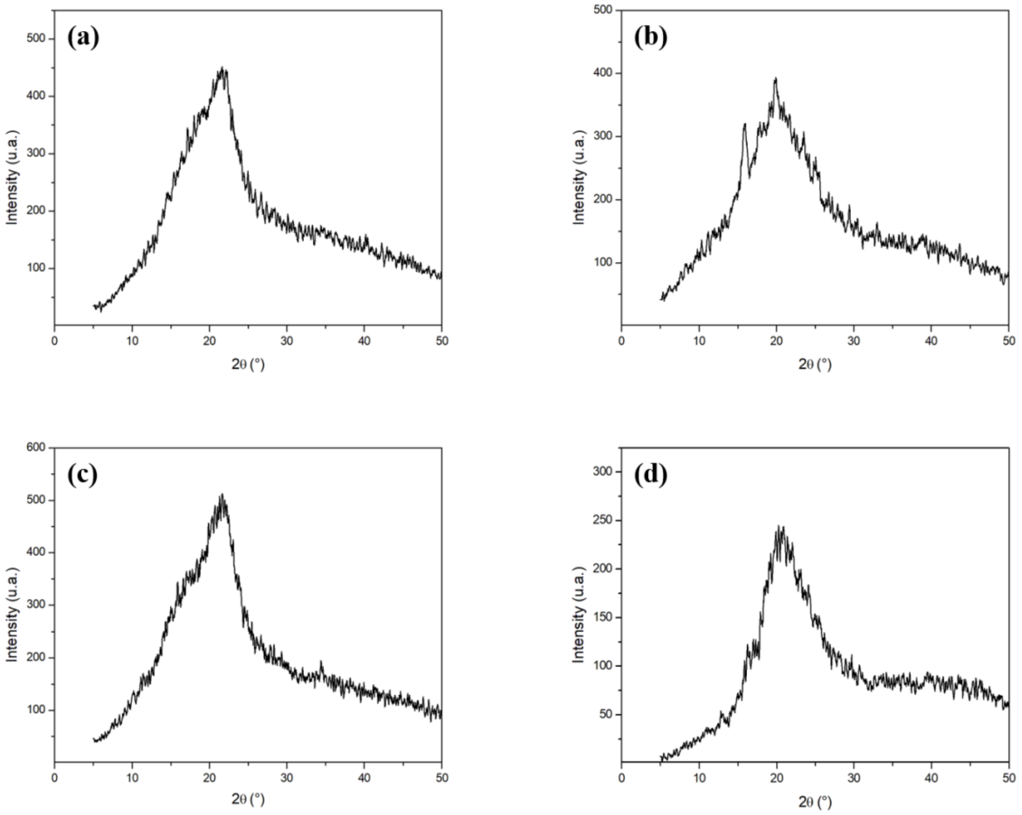

Figure S2. XRD patterns of butiá precursors (a) FIB, (b) ALM, (c) END, and (d) DOA.

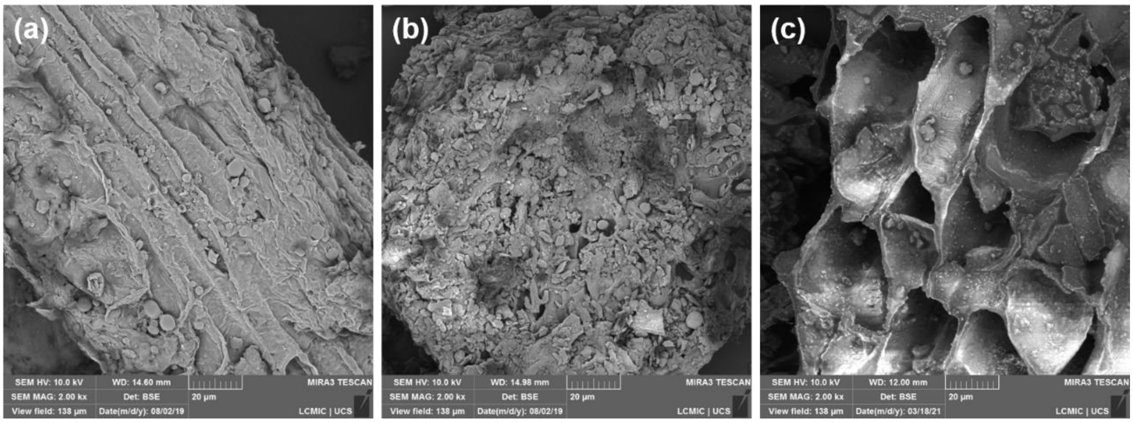

Figure S3. SEM micrographs of butiá precursors (a) FIB, (b) END and (c) DOA.

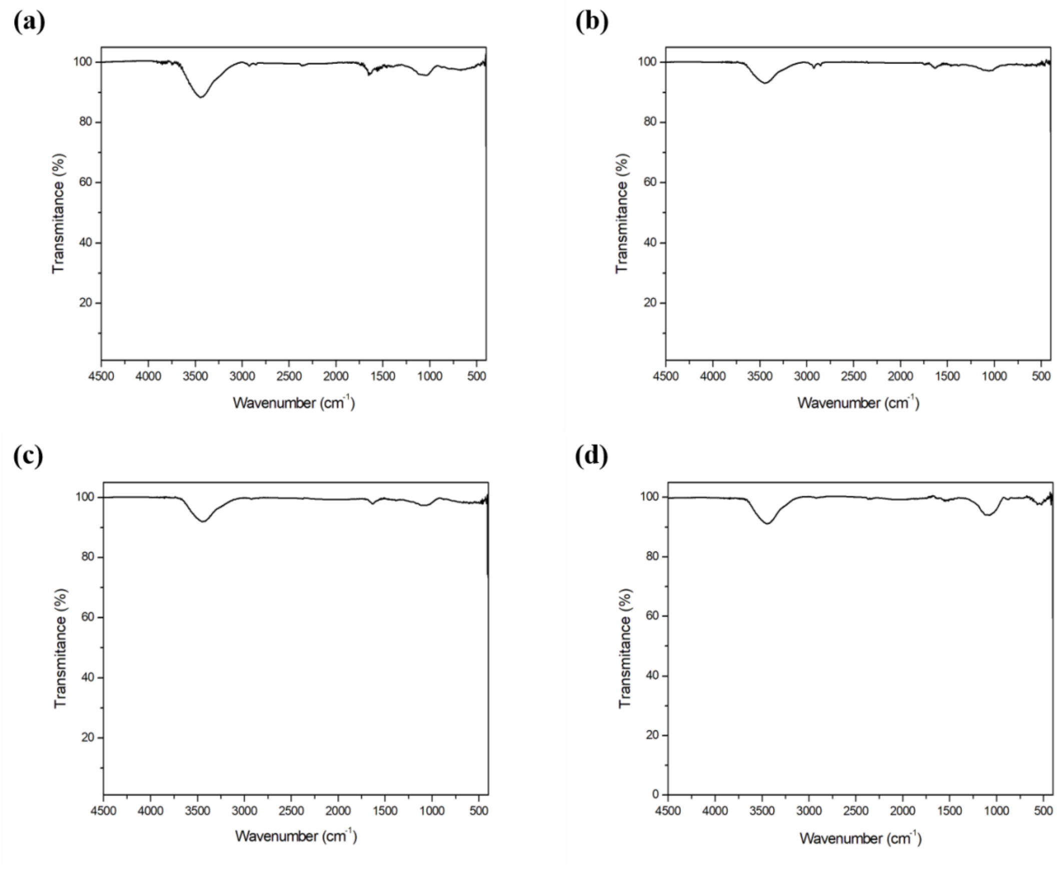

Figure S4. FTIR vibrational spectra of biochars (a) FIB.700, (b) ALM.700, (c) END.700 and (d) DOA.700.

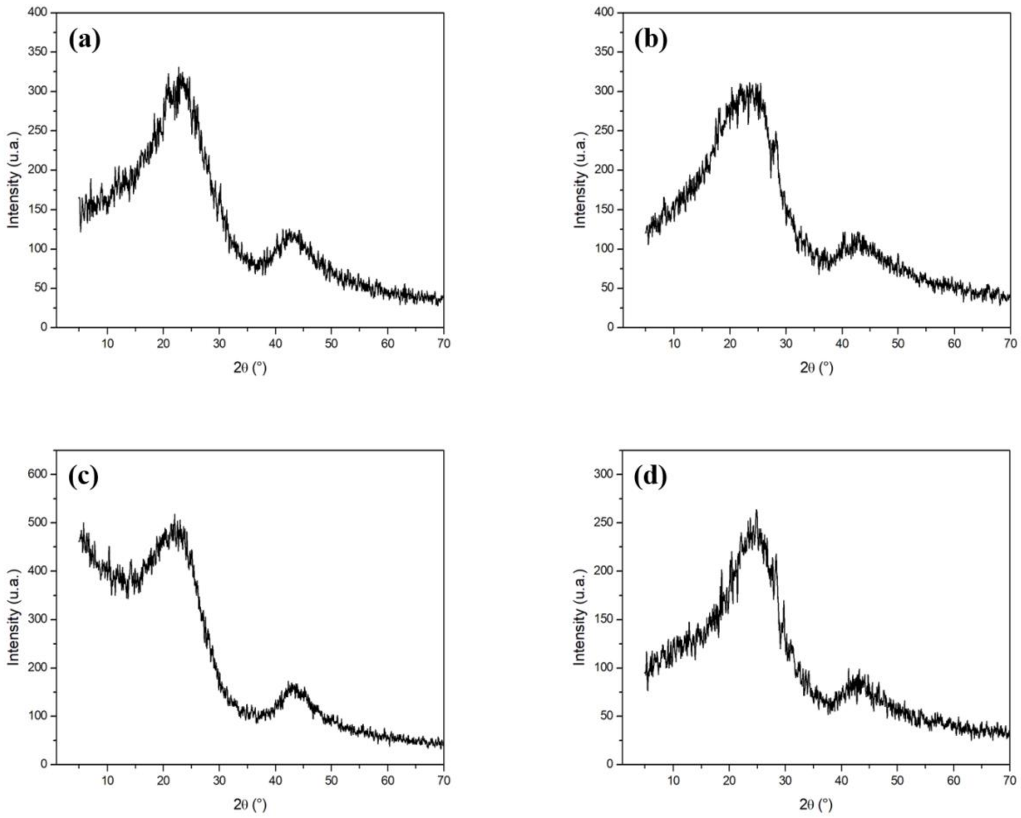

Figure S5. XRD patterns of biochars (a) FIB.700, (b) ALM.700, (c) END.700 and (d) DOA.700.

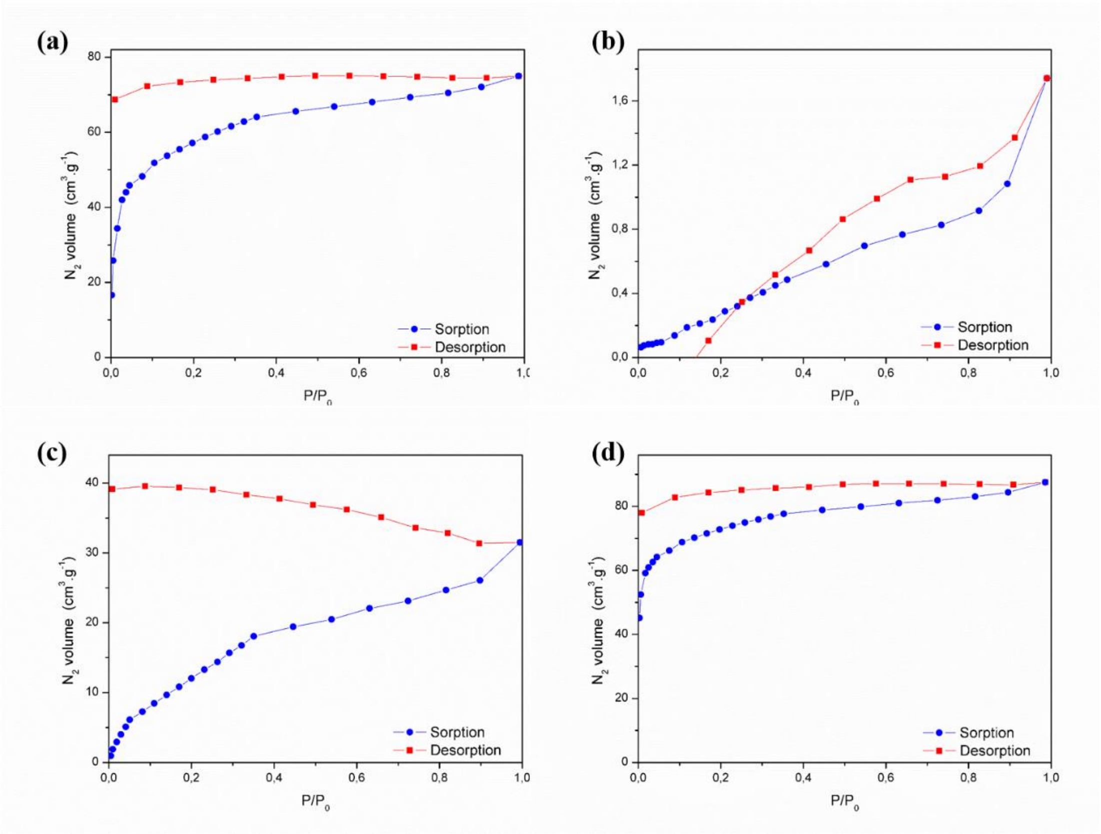

Figure S6. N_2 sorption/desorption isotherms of biochars (a) FIB.700, (b) ALM.700, (c) END.700 and (d) DOA.700.

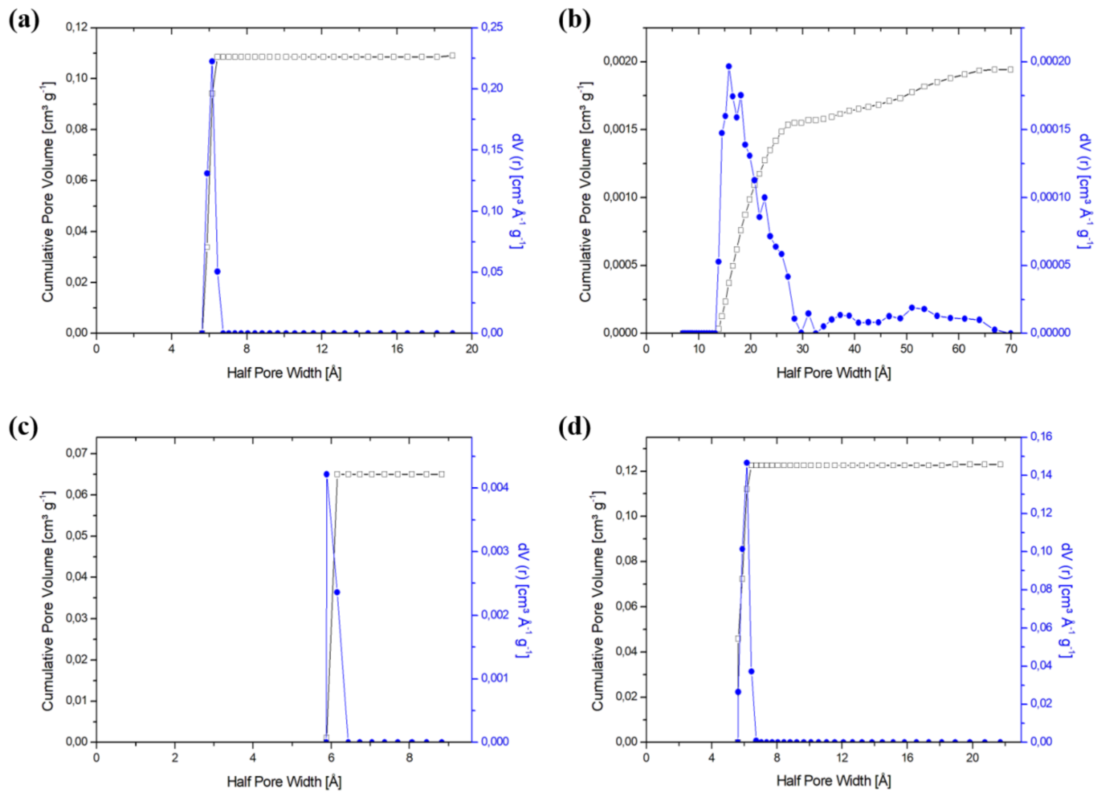

Figure S7. Pore size distribution for biochars (a) FIB.700, (b) ALM.700, (c) END.700 and (d) DOA.700.

Table S1 Surface area, CO_2 adsorption capacity and activation agent from different adsorbents presented in literature

Precursor	Activation	Surface Area (m^2 g^{-1})	CO_2 Adsorption (mg g^{-1})	CO_2 Adsorption (mg CO_2 m^{-2})	Fonte
**ALM.700**	**-**	**1.92**	**48.87**	**25.453**	**This work**
Marine shale	-	19.62	293.98	14.984	[1]
Chicken manure wastes	-	4.38	47.98	10.954	[2]
Arundo donax	-	16	87.18	5.449	[3]
Chicken manure wastes	KOH	22.22	85.82	3.862	[2]
Palm solid waste	-	24.5	73.06	2.982	[4]
Torrefied beech wood	-	36.2	70.41	1.945	[5]
**END.700**	**-**	**58.39**	**66.43**	**1.138**	**This work**
Chicken manure wastes	HCl	136.75	77.02	0.563	[2]
African palm shell	KOH	365	193.64	0.531	[6]
Kevlar carbon fibers	CO_2	469	224.45	0.479	[7]
Arundo donax	$ZnCl_2$	1420	498.33	0.351	[8]
Pollen	KOH	232	77.89	0.336	[9]
Arundo donax	KOH	849	277.26	0.327	[3]
Pomegranate peels	KOH	585	180.88	0.309	[10]
Palm kern activated carbon	CO_2	167.08	50.17	0.300	[4]
**FIB.700**	**-**	**183.59**	**54.59**	**0.297**	**This work**
Macadamia nut shell	CO_2	425	123.23	0.290	[11]
Arundo donax	KOH	637	165.39	0.260	[3]
**DOA.700**	**-**	**220.43**	**51.76**	**0.235**	**This work**
Paulownia sawdust	KOH	1643	352.08	0.214	[12]
Cokes	KOH	1375	250.86	0.182	[13]
Coal char	KOH	675.37	107.82	0.160	[14]

African palm shells	KOH	1890	277.26	0.147	[6]
Carrot peels	KOH	1379	183.96	0.133	[10]
Coconut shell	CO_2	1327	171.64	0.129	[15]
Melamine-doped phenolic-resins	KOH	1196	154.48	0.129	[16]
Paulownia sawdust	KOH	1643	211.25	0.129	[12]
Rice husk	CO_2	1097	136.43	0.124	[17]
Fern leaves	KOH	1593	181.32	0.114	[10]
CCA-treated wood	H_3PO_4/CO_2	773	83.0	0.107	[18]
Coal char	KOH	1352.06	143.03	0.106	[14]
Pollen	KOH	1460	150.51	0.103	[9]
Polyacrylonitrile	NaOH	1020	96.82	0.095	[19]
Polyacrylonitrile	$NaNH_2$	833	77.02	0.092	[19]
Beer Waste	Hidrothermal	622	57.50	0.092	[20]
Polyacrylonitrile	K_2CO_3	1250	107.38	0.086	[19]
Cotton Stalk	$Al_2(SO_4)_3$	2695	186.6	0.069	[21]
Arundo donax	$ZnCl_2$	1420	88.02	0.062	[3]
Empty palm fruit bunch	-	1080	49.29	0.046	[22]
Beer Waste	H_3PO_4	1073	35.21	0.033	[20]
Poplar Anthers	KOH	3322	89.78	0.027	[23]
Empty palm fruit bunch	Hidrothermal	1163	29.09	0.025	[22]
Empty palm fruit bunch	Hidrothermal	2510	37.58	0.015	[22]

Supplementary References

1. Du, X.; Cheng, Y.; Liu, Z.; Hou, Z.; Wu, T.; Lei, R.; Shu, C. Study on the Adsorption of CH4, CO2 and Various CH4/CO2 Mixture Gases on Shale. *Alexandria Eng. J.* **2020**, *59*, 5165–5178, doi:10.1016/j.aej.2020.09.046.
2. Yıldız, Z.; Kaya, N.; Topcu, Y.; Uzun, H. Pyrolysis and Optimization of Chicken Manure Wastes in Fluidized Bed Reactor: CO2 Capture in Activated Bio-Chars. *Process Saf. Environ. Prot.* **2019**, *130*, 297–305, doi:10.1016/j.psep.2019.08.011.
3. Singh, G.; Kim, I.Y.; Lakhi, K.S.; Srivastava, P.; Naidu, R.; Vinu, A. Single Step Synthesis of Activated Bio-Carbons with a High Surface Area and Their Excellent CO2 Adsorption Capacity. *Carbon N. Y.* **2017**, *116*, 448–455, doi:10.1016/j.carbon.2017.02.015.
4. Nasri, N.S.; Hamza, U.D.; Ismail, S.N.; Ahmed, M.M.; Mohsin, R. Assessment of Porous Carbons Derived from Sustainable Palm Solid Waste for Carbon Dioxide Capture. *J. Clean. Prod.* **2014**, *71*, 148–157, doi:10.1016/j.jclepro.2013.11.053.
5. Wedler, C.; Span, R. A Pore-Structure Dependent Kinetic Adsorption Model for Consideration in Char Conversion – Adsorption Kinetics of CO2 on Biomass Chars. *Chem. Eng. Sci.* **2021**, *231*, 116281, doi:10.1016/j.ces.2020.116281.
6. Ello, A.S.; De Souza, L.K.C.; Trokourey, A.; Jaroniec, M. Coconut Shell-Based Microporous Carbons for CO2 Capture. *Microporous Mesoporous Mater.* **2013**, *180*, 280–283, doi:10.1016/j.micromeso.2013.07.008.
7. Conte, G.; Stelitano, S.; Policicchio, A.; Minuto, F.D.; Lazzaroli, V.; Galiano, F.; Agostino, R.G. Assessment of Activated Carbon Fibers from Commercial Kevlar® as Nanostructured Material for Gas Storage: Effect of Activation Procedure and Adsorption of CO2 and CH4. *J. Anal. Appl. Pyrolysis* **2020**, *152*, 104974, doi:10.1016/j.jaap.2020.104974.
8. Singh, G.; Kim, I.Y.; Lakhi, K.S.; Joseph, S.; Srivastava, P.; Naidu, R.; Vinu, A. Heteroatom Functionalized Activated Porous Biocarbons and Their Excellent Performance for CO2 Capture at High Pressure. *J. Mater. Chem. A* **2017**, *5*, 21196–21204, doi:10.1039/c7ta07186h.
9. Choi, S.W.; Tang, J.; Pol, V.G.; Lee, K.B. Pollen-Derived Porous Carbon by KOH Activation: Effect of Physicochemical Structure on CO 2 Adsorption. *J. CO2 Util.* **2019**, *29*, 146–155, doi:10.1016/j.jcou.2018.12.005.
10. Serafin, J.; Narkiewicz, U.; Morawski, A.W.; Wróbel, R.J.; Michalkiewicz, B. Highly Microporous Activated Carbons from Biomass for CO2 Capture and Effective Micropores at Different Conditions. *J. CO2 Util.* **2017**, *18*, 73–79, doi:10.1016/j.jcou.2017.01.006.
11. Bae, J.S.; Su, S. Macadamia Nut Shell-Derived Carbon Composites for Post Combustion CO2 Capture. *Int. J. Greenh. Gas Control* **2013**, *19*, 174–182, doi:10.1016/j.ijggc.2013.08.013.
12. Zhu, X.L.; Wang, P.Y.; Peng, C.; Yang, J.; Yan, X. Bin Activated Carbon Produced from Paulownia Sawdust for High-Performance CO2 Sorbents. *Chinese Chem. Lett.* **2014**, *25*, 929–932, doi:10.1016/j.cclet.2014.03.039.
13. Labus, K.; Gryglewicz, S.; Machnikowski, J. Granular KOH-Activated Carbons from Coal-Based Cokes and Their CO 2 Adsorption Capacity. *Fuel* **2014**, *118*, 9–15, doi:10.1016/j.fuel.2013.10.042.
14. Quan, C.; Wang, H.; Jia, X.; Gao, N. Effect of Carbonization Temperature on CO2 Adsorption Behavior of Activated Coal Char. *J. Energy Inst.* **2021**, *97*, 92–99, doi:10.1016/j.joei.2021.04.003.
15. Ello, A.S.; De Souza, L.K.C.; Trokourey, A.; Jaroniec, M. Development of Microporous Carbons for CO2 Capture by KOH Activation of African Palm Shells. *J. CO2 Util.* **2013**, *2*, 35–38, doi:10.1016/j.jcou.2013.07.003.
16. Cong, H.; Zhang, M.; Chen, Y.; Chen, K.; Hao, Y.; Zhao, Y.; Feng, L. Highly Selective CO2 Capture by Nitrogen Enriched Porous Carbons. *Carbon N. Y.* **2015**, *92*, 297–304, doi:10.1016/j.carbon.2015.04.052.
17. Li, M.; Xiao, R. Preparation of a Dual Pore Structure Activated Carbon from Rice Husk Char as an Adsorbent for CO2 Capture. *Fuel Process. Technol.* **2019**, *186*, 35–39, doi:10.1016/j.fuproc.2018.12.015.
18. Botomé, M.L.; Poletto, P.; Junges, J.; Perondi, D.; Dettmer, A.; Godinho, M. Preparation and Characterization of a Metal-Rich Activated Carbon from CCA-Treated Wood for CO2 Capture. *Chem. Eng. J.* **2017**, *321*, 614–621, doi:10.1016/j.cej.2017.04.004.
19. Singh, J.; Basu, S.; Bhunia, H. CO2 Capture by Modified Porous Carbon Adsorbents: Effect of Various Activating Agents. *J. Taiwan Inst. Chem. Eng.* **2019**, *102*, 438–447, doi:10.1016/j.jtice.2019.06.011.
20. Hao, W.; Björkman, E.; Lilliestråle, M.; Hedin, N. Activated Carbons Prepared from Hydrothermally Carbonized Waste Biomass Used as Adsorbents for CO2. *Appl. Energy* **2013**, *112*, 526–532, doi:10.1016/j.apenergy.2013.02.028.
21. Pramanik, P.; Patel, H.; Charola, S.; Neogi, S.; Maiti, S. High Surface Area Porous Carbon from Cotton Stalk Agro-Residue for CO2adsorption and Study of Techno-Economic Viability of Commercial Production. *J. CO2 Util.* **2021**, *45*, 101450, doi:10.1016/j.jcou.2021.101450.
22. Parshetti, G.K.; Chowdhury, S.; Balasubramanian, R. Biomass Derived Low-Cost Microporous Adsorbents for Efficient CO2 Capture. *Fuel* **2015**, *148*, 246–254, doi:10.1016/j.fuel.2015.01.032.
23. Song, J.; Shen, W.; Wang, J.; Fan, W. Superior Carbon-Based CO2 Adsorbents Prepared from Poplar Anthers. *Carbon N. Y.* **2014**, *69*, 255–263, doi:10.1016/j.carbon.2013.12.024.
